# Constructing majority-rule supertrees

**DOI:** 10.1186/1748-7188-5-2

**Published:** 2010-01-04

**Authors:** Jianrong Dong, David Fernández-Baca, FR McMorris

**Affiliations:** 1 Department of Computer Science, Iowa State University, Ames, IA 50011, USA; 2 Department of Applied Mathematics, Illinois Institute of Technology, Chicago, IL 60616, USA

## Abstract

**Background:**

Supertree methods combine the phylogenetic information from multiple partially-overlapping trees into a larger phylogenetic tree called a supertree. Several supertree construction methods have been proposed to date, but most of these are not designed with any specific properties in mind. Recently, Cotton and Wilkinson proposed extensions of the majority-rule consensus tree method to the supertree setting that inherit many of the appealing properties of the former.

**Results:**

We study a variant of one of Cotton and Wilkinson's methods, called majority-rule (+) supertrees. After proving that a key underlying problem for constructing majority-rule (+) supertrees is NP-hard, we develop a polynomial-size exact integer linear programming formulation of the problem. We then present a data reduction heuristic that identifies smaller subproblems that can be solved independently. While this technique is not guaranteed to produce optimal solutions, it can achieve substantial problem-size reduction. Finally, we report on a computational study of our approach on various real data sets, including the 121-taxon, 7-tree Seabirds data set of Kennedy and Page.

**Conclusions:**

The results indicate that our exact method is computationally feasible for moderately large inputs. For larger inputs, our data reduction heuristic makes it feasible to tackle problems that are well beyond the range of the basic integer programming approach. Comparisons between the results obtained by our heuristic and exact solutions indicate that the heuristic produces good answers. Our results also suggest that the majority-rule (+) approach, in both its basic form and with data reduction, yields biologically meaningful phylogenies.

## Background

### Introduction

A supertree method begins with a collection of phylogenetic trees with possibly different leaf (taxon) sets, and assembles them into a larger phylogenetic tree, a *supertree*, whose taxon set is the union of the taxon sets of the input trees. Interest in supertrees was sparked by Gordon's paper [1]. Since then, particularly during the past decade, there has been a flurry of activity with many supertree methods proposed and studied from the algorithmic, theoretical, and biological points of view. The appeal of supertree synthesis is that it can combine disparate data to provide a high-level perspective that is harder to attain from individual trees. A recent example of the use of this approach is the species-level phylogeny of nearly all extant Mammalia constructed by Bininda-Emonds [2] from over 2,500 partial estimates. Several of the known supertree methods are reviewed in the book edited by Bininda-Emonds [3] — more recent papers with good bibliographies include [4,5]. There is still much debate about what specific properties should (can), or should not (cannot), be satisfied by supertree methods. Indeed, it is often a challenging problem to rigorously determine the properties of a supertree method that gives seemingly good results on data, but is heuristic.

The well-studied consensus tree problem can be viewed as the special case of the supertree problem where the input trees have identical leaf sets. Consensus methods in systematics date back to [6]; since then, many consensus methods have been designed. A good survey of these methods, their properties, and their interrelationships is given by Bryant [7], while the axiomatic approach and the motivation from the social sciences is found in Day and McMorris' book [8]. One of the most widely used methods is the majority-rule consensus tree [9,10], which is the tree that contains the splits displayed by the majority of the input trees. Not only does this tree always exist, but it is also unique, can be efficiently constructed [11], and has the property of being a *median tree *relative to the symmetric-difference distance (also known as the Robinson-Foulds distance [12,13]). That is, the majority-rule consensus tree is a tree whose total Robinson-Foulds distance to the input trees is minimum.

The appealing qualities of the majority-rule consensus method have made it attractive to try to extend the method to the supertree setting, while retaining as many of its good characteristics as possible. Cotton and Wilkinson [14] were able to define two such extensions (despite some doubts about whether such an extension was possible [15]) and at least two additional ones have been studied since [16]. Here we study one of the latter variants, called *graft-refine majority-rule (+) supertrees *in [16], and here simply referred to as *majority-rule (+) supertrees*. These supertrees satisfy certain desirable properties with respect to what information from the input trees, in the form of splits, is displayed by them (see the Preliminaries). The key idea in this method is to expand the input trees by grafting leaves onto them to produce trees over the same leaf set. The expansion is done so as to minimize the distance from the expanded trees to their median relative to the Robinson-Foulds distance. The supertree returned is the strict consensus of the median trees with minimum distance to the expanded input trees; these median trees are called *optimal candidate supertrees*.

After showing that computing an optimal candidate supertree is NP-hard, we develop a characterization of these supertrees that allows us to formulate the problem as a polynomial-size integer linear program (ILP). We then describe an implementation that enables us to solve moderately large problems exactly. We show that, in practice, the majority-rule (+) supertree can be constructed quickly once an optimal candidate supertree has been identified. Furthermore, we observe that the supertrees produced are similar to biologically reasonable trees, adding further justification to the majority-rule (+) approach.

In addition to the exact ILP formulation, we also introduce a data reduction heuristic that identifies "reducible" sets of taxa. Informally, these are taxa that are clustered in the same way by all the input trees. By restricting the original profile to the taxa in any such set, we get a "satellite profile" that can be much smaller than the original one. At the same time, the original profile can be reduced by representing all the taxa in the set by a single supertaxon. A supertree for the original profile is obtained by solving each of these supertree problems independently and combining the answers. This approach allows us to tackle supertree problems that are well beyond the limits of the basic ILP method. Thus, whereas the latter allowed us to solve instances at most 40 taxa, the former enabled us to handle the Seabirds data set of Kennedy and Page [17], which as 121 taxa. While the data reduction technique is not guaranteed to produce the same answers as the exact method, we present empirical evidence that it produces good results. Moreover, reducible sets often correspond to meaningful biological classification units that likely should be respected by any supertree.

We should mention that the supertree method most commonly used in practice is matrix representation with parsimony (MRP) [18,19]. MRP first encodes the input trees as incomplete binary characters, and then builds a maximum-parsimony tree for this data. The popularity of MRP is perhaps due to the widespread acceptance of the philosophy underlying parsimony approaches and the availability of excellent parsimony software (e.g., [20]). However, while parsimony is relatively easy to justify in the original tree-building problem (in which homoplasy represents additional assumptions of evolutionary changes) a justification for its use as a supertree construction method is not quite as obvious. Perhaps the main criticism of MRP, as well as other tree construction methods, is that it can produce unsupported groups [21,22]. The provable properties of majority-rule (+) supertrees [14,16] prevent such anomalies. There has been previous work on ILP in phylogenetics, much of it dealing with parsimony or its relative, compatibility [23-27]. Our work uses some of these ideas (especially those of [26]), but the context and the objective function are quite different. In particular, the need to handle all possible expansions of the input trees necessitates the introduction of new techniques.

### Preliminaries

#### Basic definitions and notation

Our terminology largely follows [28]. A *phylogenetic tree *is an unrooted leaf-labeled tree where every internal node has degree at least three. We will use "tree" and "phylogenetic tree" interchangeably. The leaf set of a tree *T *is denoted by ℒ(*T*).

A *profile *is a tuple of trees *P *= (*t*_1_,..., *t*_*k*_). Each *t*_*i *_in *P *is called an *input tree*. Let ℒ(*P*) = ∪_*i*∈*K*_ℒ(*t*_*i*_), where *K *denotes the set {1,..., *k*}. An input tree *t*_*i *_is *plenary *if ℒ(*t*_*i*_) = ℒ(*P*). Tree *T *is a *supertree *for profile *P *if ℒ(*T*) = ℒ(*P*).

A *split *is a bipartition of a set. We write *A*|*B *to denote the split whose parts are *A *and *B*. The order here does not matter, so *A*|*B *is the same as *B*|*A*. Split *A*|*B *is *nontrivial *if each of *A *and *B *has at least two elements; otherwise it is *trivial*. Split *A|B extends *another split *C*|*D *if *A *⊇ *C *and *B *⊇ *D*, or *A *⊇ *D *and *B *⊇ *C*.

Phylogenetic tree *T displays *split *A*|*B *if there is an edge in *T *whose removal gives trees *T*_1 _and *T*_2 _such that *A *⊆ ℒ(*T*_1_) and *B *⊆ ℒ(*T*_2_). A split *A*|*B *is *full *with respect to a tree *T *if *A *∪ *B *= ℒ(*T*); *A*|*B *is *partial *with respect to *T *if *A *∪ *B *⊂ ℒ(*T*). Split *A*|*B *is *plenary *with respect to a profile *P *if *A *∪ *B *= ℒ(*P*). The set of all nontrivial full splits displayed by *T *is denoted Spl(*T*). It is well known that the full splits of *T *uniquely identify *T *[[28], p. 44]. Let *S *⊆ ℒ(*T*). The *restriction of T to S*, denoted *T*|*S*, is the phylogenetic tree with leaf set *S *such that

Let *T' *be a phylogenetic tree such that *S *= ℒ(*T'*) ⊆ ℒ(*T*). Then, *T displays T' *if Spl(*T'*) ⊆ Spl(*T*|*S*).

A set of splits is *compatible *if there is a tree *T *that displays them all. Tree *T *is *compatible with *a set of splits  if there is a tree *T' *that displays *T *and .

Let *T*_1 _and *T*_2 _be two phylogenetic trees over the same leaf set. The *symmetric-difference distance*, also known as *Robinson-Foulds distance *[13], between *T*_1 _and *T*_2_, denoted *d*(*T*_1_, *T*_2_), is defined as(1)

The *majority splits *in a profile *P *= (*t*_1_,..., *t*_*k*_) are the splits displayed by more than  of the input trees. A *majority plenary split *is a plenary split that is also a majority split. Similarly, a *majority partial split *is a partial split that is also a majority split.

*Rooted *phylogenetic trees can be viewed as a special case of unrooted trees. That is, we can view a profile of rooted trees as unrooted trees, all of which have a common taxon called the root. Thus, in a split in a rooted tree, one of the two parts must contain the root; the part that does not contain the root is called a *cluster *(or *clade*, or *monophyletic group*). All of the above concepts (eg., compatibility and distance), as well as those introduced in the rest of this paper, directly apply to rooted trees. However, we shall not elaborate on this here.

To close this section, we examine the *consensus problem*, the special case of the supertree problem where the profile *P *= (*T*_1_,..., *T*_*k*_) consists of trees that have the same leaf set. The *strict consensus *of *P *is the tree that displays exactly the plenary splits present in every tree in the profile. The *majority-rule consensus tree *of *P*, denoted Maj(*P*), is the tree that displays all the majority plenary splits in *P *[10]. For any phylogeny *T *with ℒ(*T*) = ℒ(*P*), define the *distance *from *T *to *P *as dist(*T*, *P*) = Σ_*i*∈*K*_*d*(*T*, *T*_*i*_), where *d *denotes the symmetric-difference distance. Any *T *with leaf set ℒ(*P*) that minimizes dist(*T*, *P*) is called a *median tree *for *P*. It is known that Maj(*P*) is a median tree for *P*; indeed, it follows from [9] that Maj(*P*) is the strict consensus of the median trees for *P*. The (median) *score *of *P *is defined as *s*(*P*) = min_*T*:ℒ(*T*)=ℒ(*P*) _dist(*T*, *P*). Thus, *s*(*P*) = dist(Maj(*P*), *P*).

### Majority-rule (+) supertrees

Here we describe a variant (suggested by Bill Day) of one of Cotton and Wilkinson's [14] extensions of majority-rule consensus to the supertree setting.

The *span *of an input tree *t*, denoted by ⟨*t*⟩, is the set of all trees on ℒ(*P*) that display *t*. The *span *of a profile *P *= (*t*_1_,..., *t*_*k*_), denoted ⟨*P*⟩, is the set of all *k*-tuples *R *= (*T*_1_,..., *T*_*k*_), where *T*_*i *_∈ ⟨*t*_*i*_⟩ for each *i *∈ *K*. Each *R *∈ ⟨*P*⟩ is called a *representative selection *for *P *and Maj(*R*) is called a *candidate supertree*.

An *optimal representative selection *is a representative selection *R *with minimum score *s*(*R*) over all *R *∈ ⟨*P*⟩. We refer to Maj(*R*) as the *optimal candidate supertree *associated with *R*. The *majority-rule (+) supertree *of profile *P*, denoted by Maj^+^(*P*), is the strict consensus of all the optimal candidate supertrees. We have shown elsewhere [16] that Maj^+^(*P*) satisfies the following appealing properties (originally conjectured by Cotton and Wilkinson).

(CW1) Maj^+^(*P*) displays all of the majority plenary splits in *P*.

(CW2) Maj^+^(*P*) is compatible with each majority partial split in *P*.

(CW3) Each split in Maj^+^(*P*) is compatible with a majority of the trees in *P*.

(CW4) Every plenary split in Maj^+^(*P*) extends at least one input tree full split.

We should note that majority-rule (+) supertrees, as defined above, do not generalize majority-rule consensus. That is, when used in the consensus setting, Maj^+^(*P*) is not, in general, the same as Maj(*P*). Nevertheless, majority-rule (+) consensus trees have a simple characterization that yields an efficient algorithm for computing them (see Theorem 1 of the Methods).

The majority-rule (+) supertrees we study differ from other variants in the way the span of an input tree is defined. Cotton and Wilkinson originally defined the span of a tree *t *as the set of all plenary *binary *trees that display *t *[14]. This version does not generalize majority-rule consensus and does not satisfy (CW4) [16]. In a more recent version, suggested by Wilkinson (personal communication), the span of *t *is the set of all plenary trees *T *such that *T*|ℒ(*t*) = *t*. This definition of span prohibits refinement of any original polytomies (nodes of degree at least four) in *t*. It can be shown that the supertree method that results from using this definition generalizes majority-rule consensus, and that it satisfies properties (CW1)-(CW4) [16]. Nonetheless, we have preferred Day's variant for two reasons. First, we have found it computationally easier to deal with than the others. More importantly, it can be argued that a strict generalization of majority-rule consensus might not be the ideal approach for supertree construction: In practice, one often encounters profiles where different trees "specialize" in different groups of taxa, leaving other groups largely unresolved or unrepresented. In a combined analysis, each input tree should contribute its own specialized information so that, jointly, the trees lead to a well-resolved supertree. A strict generalization of majority rule would disallow this, since the method discards minority information. In contrast, the majority-rule (+) supertrees presented here preserve this fine-grained information, unless it were substantially contradicted by the remaining trees (the sense in which this is true can be gleaned from Theorem 1 of the Methods).

## Methods

### Constructing optimal candidate supertrees

We first consider the consensus version of the problem. Let *P *= (*T*_1_,..., *T*_*k*_) be a profile of trees over the same leaf set. Given a plenary split *X *= *A*|*B*, define

and

The theorem below, proved elsewhere (Dong, Fernández-Baca, McMorris, and Powers: Majority-rule (+) consensus trees, *unpublished*), characterizes the majority-rule (+) consensus tree of a profile and implies that this tree can be computed in polynomial time.

**Theorem 1**. *For any profile P*, Maj^+^(*P*) *is precisely the tree that displays every split X such that *. *Furthermore*, Maj^+^(*P*) *is an optimal candidate tree for P, as well as the strict consensus of all optimal candidate trees for P*.

On the other hand, the next result suggests that finding the majority-rule (+) supertree for a profile of trees with partially overlapping leaf sets may be hard.

**Theorem 2**. *There is no polynomial-time algorithm to construct an optimal candidate supertree unless P = NP*.

*Proof*. We show that if there is a polynomial time algorithm to compute an optimal candidate supertree, then there exists a polynomial-time algorithm for the quartet compatibility problem, which is known to be NP-complete [29]. The quartet compatibility problem asks whether, given a collection *Q *of trees on four leaves, there exists a single tree that displays them all. If the answer is "yes", we say that *Q *is *compatible*. Let *Q *be an instance of quartet compatibility. Construct a profile *P *that simply consists of the trees in *Q *in some arbitrary order. We claim that *Q *is compatible if and only if *P *has an optimal candidate supertree with a score of zero. Suppose first that *Q *is compatible and that *T *is any tree that displays each element of *Q*. Then, for each tree *t *in *P*, *T *∈ ⟨*t*⟩, because all the splits in *T *must be compatible with *t*, so any split in *T *that is not in *t *can be added to *t*. Hence, *T *is a candidate tree for *P *with a score of zero, and thus *T *is also an optimal candidate supertree. Conversely, if *P *has an optimal candidate supertree with zero score, it can be seen that *T *displays all the quartets in *Q*; i.e., *Q *is compatible.

In the next sections, we show that despite the above result, moderately large majority-rule (+) supertree problems can be solved using integer linear programming. For this, we need to address a potential complication: Since the definition of ⟨*t*⟩ allows refinement of multifurcations in *t*, a tree *T *∈ ⟨*t*⟩ can contain many more nontrivial splits than *t*; indeed, we cannot predetermine the number of nontrivial splits *T *will contain. We circumvent this potential problem by defining a restricted version of the span.

Given an input tree *t *in a profile *P*, the *restricted span *of *t*, denoted ⟨*t*⟩_*r *_is the set of all plenary trees *T *such that every nontrivial split in *T *extends a distinct nontrivial split in *t*. Thus, *|*Spl(*T*)*| *= *|*Spl(*t*)*|*. Note that *T *is obtained from *t *by filling in each of *t*'s splits, by adding zero or more taxa to each part, to make them plenary splits in such a way that the resulting splits are compatible. Note also that ⟨*t*⟩_*r *_⊆ ⟨*t*⟩. The *restricted span *of a profile *P *= (*t*_1_,..., *t*_*k*_), denoted ⟨*P*⟩_*r *_is the set of all *R *= (*T*_1_,..., *T*_*k*_) for *P *such that *T*_*i *_∈ ⟨*t*⟩_*r *_for each *i *∈ *K*. Each *R *∈ ⟨*P*⟩_*r *_is called a *restricted representative selection *for *P*. Since ⟨*P*⟩_*r *_⊆ ⟨*P*⟩, the restricted span represents an intermediate level between the input profile and the original definition of span. The restricted span is more manageable than the original span because it does not allow any refinement of input trees. In the rest of this section, we will show how to obtain majority-rule (+) supertrees directly from the restricted span.

Before presenting the first of the two main results of this section, we need to introduce some new concepts. An optimal candidate supertree *T *for a profile *P *is *minimal *if contracting any edge in *T *yields a tree that is not an optimal candidate supertree. Let *R *= (*T*_1_,..., *T*_*k*_) and  be two representative selections for a profile *P*. We say that *R displays R' *if *T*_*i *_displays  for every *i *∈ *K*. Theorem 1 motivates the next definition. The *completion *of a representative selection *R *= (*T*_1_,..., *T*_*k*_) for a profile *P *is the representative selection  obtained as follows: For every *i *∈ *K*,  is the tree constructed by inserting into *T*_*i *_each plenary split *X *= *A*|*B *compatible with *T*_*i *_such that .

**Theorem 3**. *Let T be a minimal optimal candidate supertree for a profile P and let R *∈ ⟨*P*⟩ *be such that T *= Maj(*R*). *Consider any G *∈ ⟨*P*⟩_*r *_*such that G is displayed by R. Then, R is the completion of G and T *= Maj^+^(*G*).

*Proof*. We begin by proving that *T *is an optimal candidate supertree for *G*. Assume the contrary. Then, there exists another candidate tree *T' *for *G *such that (i) *T' *= Maj(*R'*) for some *R' *∈ ⟨*G*⟩ and (ii) *s*(*R'*) <*s*(*R*). But then, since ⟨*G*⟩ ⊆ ⟨*P*⟩, we have *R' *∈ ⟨*P*⟩, and thus (ii) contradicts the optimality of *T *for *P*.

Next, we argue that *T *is a *minimal *optimal candidate supertree for profile *G*. Suppose this is not true. Then, *T *displays an optimal candidate supertree *T' *for *G *such that *T *≠ *T'*. Consider any *R' *∈ ⟨*G*⟩ such that *T' *= Maj(*R'*). Since *T *and *T' *are both optimal for *G*, *s*(*R*) = *s*(*R'*). Since *R' *displays *P*, we have *R' *∈ ⟨*P*⟩. Hence, *T' *is also an optimal candidate supertree for *P*. This, however, contradicts the assumption that *T *is a minimal optimal candidate tree for *P*.

By Theorem 1, Maj^+^(*G*) is an optimal candidate supertree for *G*, as well as the strict consensus of all optimal candidate supertrees for *G*. Therefore, Maj^+^(*G*) is the only minimal optimal candidate supertree for *G*. Hence *T *= Maj^+^(*G*).

Suppose *R *= (*T*_1_,..., *T*_*k*_) and let  be the completion of *G*. We claim that  = *R*. Assume, on the contrary, that there is some *i *∈ *K *such that  ≠ *T*_*i*_. That is, , where  = Spl()\Spl(*T*_*i*_) and  = Spl(*T*_*i*_)\Spl(). Set  consists of splits *X *such that  and  consists of splits *Y *such that . By Theorem 1, *T *= Maj^+^(*G*) displays all splits *X *such that . Thus, *d*(*T*, ) <*d*(*T*, *T*_*i*_). As we are assuming that there is at least one such *i *∈ *K*, we have Σ_*i*∈*K*_*d*(*T*, ) < Σ_*i*∈*K*_*d*(*T*, *T*_*i*_), contradicting the fact that *T *is a minimal optimal candidate supertree for *G*.

Motivated by Theorem 3, we define the *adjusted score *of a representative selection *R *for a profile *P*, denoted (*R*), to be the score of the completion  of *R*; i.e., . Recall that .

**Theorem 4**. *Let P be a profile. Define * = {*G *∈ ⟨*P*⟩_*r*_:(*G*) *is minimum*} *and S *= {*T *= Maj^+^(*G*) : *G *∈ }. *Then*, Maj^+^(*P*) *is the strict consensus of *.

*Proof*. Let  be the set of all optimal candidate supertrees for *P *and let ℳ be the set of all minimal optimal candidate supertrees of *P*. In what follows, we show that . This immediately implies the theorem, because not only is (by definition) Maj^+^(*P*) the strict consensus of , but it must also be the strict consensus of ℳ.

Suppose *T *∈ ℳ. We claim that *T *∈  and, therefore, that . Let *R *be a representative selection for *P *such that *T *= Maj(*R*). Let *G *be any restricted representative selection for *P *displayed by *R*. By Theorem 3, *T *= Maj^+^(*G*) and *R *is the completion of *G*. We claim that *G *∈ ; i.e., (*G*) is minimum. Assume, by way of contradiction, that there is another *G' *∈ ⟨*P*⟩_*r *_such that . Let *R' *be the completion of *G'*. Then, , which contradicts the assumption that *T *is optimal. Therefore, (*G*) is minimum and *T *∈ .

Suppose *T *∈ . We claim that *T *∈  and, therefore, that . Let *G *∈ ⟨*P*⟩_*r *_be such that *T *= Maj+(*G*) and the adjusted score (*G*) is minimum. Let *R *be the completion of *G*. Assume, by way of contradiction, that *T *∉ . Then there is a *T' *∈ ℳ such that, if *R' *is a representative selection for *P *where *T' *= Maj(*R'*), then *s*(*R'*) <*s*(*R*). By Theorem 3, there is a *G' *∈ ⟨*P*⟩_*r *_such that *T' *= Maj^+^(*G'*) and (*G'*) = *s*(*R'*). Then (*G'*) = *s*(*R'*) <*s*(*R*) = (*G*). This contradicts the assumption that (*G*) is minimum.

### ILP formulation

In this section we first describe an ILP formulation of the optimal candidate supertree problem based on Theorem 4. The optimum solution to this ILP is a *G *∈ ⟨*P*⟩_*r *_with minimum adjusted score. For ease of exposition, we divide the variables of our ILP into three categories: *fill-in variables*, which represent the way taxa are added to the input trees to create *G*; *objective function variables*, which are used to express (*G*); and *auxiliary variables*, which are used to establish a connection between the fill-in and objective function variables. All variables are binary. After presenting our ILP model, we discuss how to use it to generate Maj^+^(*P*).

#### Fill-in variables

At the core of our ILP formulation is a matrix representation of the input trees similar to that used in MRP [18,19]. Let *P *= (*t*_1_,..., *t*_*k*_) be a profile where |ℒ(*P*)| = *n*. Assume input tree *t*_*j *_has *m*_*j *_nontrivial splits, which are assumed to be ordered in some fixed but arbitrary way. A *matrix representation *of *t*_*j *_is a *n *× *m*_*j *_matrix *M*(*t*_*j*_) whose columns are in one to one correspondence with the nontrivial splits of *t*_*j*_.

Suppose column *i *of *M*(*t*_*j*_) corresponds to split *A*|*B *in *t*_*j *_and let *x *be a taxon in ℒ(*P*). Then, *M*_*x*, *i*_(*t*_*j*_) = 1 if *x *∈ *A*, *M*_*x*, *i*_(*t*_*j*_) = 0 if *x *∈ *B*, and *M*_*x*, *i*_(*t*_*j*_) =? otherwise. We note that for unrooted trees the assignment of 1 to the *A *side of the split and of 0 to the *B *side is arbitrary. For rooted trees, all taxa in the side of a split that contains the root are assigned a 1.

Let *m *= Σ_*j*∈*K*_*m*_*j*_. A *matrix representation *of *P*, denoted *M *(*P*), is a *n *× *m *matrix *M *(*P*) obtained by concatenating matrices *M*(*t*_1_), *M*(*t*_2_),..., *M*(*t*_*k*_).

A *fill-in *of matrix *M*(*P*) is a matrix representation for a restricted representative selection *G *for *P*. Note that *M*(*G*) has no question marks and that, for every taxon *x *and split *i *such that *M*_*xi*_(*P*) ∈ {0, 1}, we have *M*_*xi*_(*G*) = *M*_*xi*_(*P*). To represent fill-ins of *M*(*P*), the ILP associates a *fill-in variable F*_*xi *_with each *x *and *i*. If *M*_*xi*_(*P*) ∈ {0, 1}, then *F*_*xi *_= *M*_*xi*_(*P*); i.e., *F*_*xi *_is fixed. If *M*_*xi*_(*P*) =?, *F*_*xi *_will be assigned a value of 0 or 1, representing an assignment of taxon *x *to one of the two sides of split *i*. Our ILP has constraints (described below) to ensure that each value assignment to the *F*-variables corresponds to a restricted representative selection for *P*. That is, there must exist a *G *∈ ⟨*P*⟩_*r *_such that *M*_*xi*_(*G*) = *F*_*xi *_for every *x *and *i*.

#### Objective function variables

The objective is to minimize (*G*) over all *G *∈ ⟨*P*⟩_*r*_, where each *G *is represented by a fill-in of *M*(*P*). By definition, (*G*) = dist(Maj^+^(*G*), *R*), where  is the completion of *G *= (*T*_1_,..., *T*_*k*_). We do not, however, construct Maj^+^(*G*) and *R *explicitly. Instead, we proceed indirectly, using the fact that, by Theorems 1 and 3, all splits in Maj^+^(*G*) and *R *are already in *G*. Indeed, those theorems and the definition of Robinson-Foulds distance (Equation 1) imply that(2)

The next result, which follows Theorems 1 and 3, allows us to count directly from *G *the contribution of each split *X *∈ Spl(Maj^+^(*G*)) ∪ Spl() to *d*(Maj^+^(*G*), ).

**Lemma 1**. *Let P be a profile and suppose G *∈ ⟨*P*⟩_*r*_. *Then, for each j *∈ *K*,

*(i) X *∈ Spl(Maj^+^(*G*))\Spl() *if and only if **and j *∈ .

*(ii) X *∈ Spl()\Spl(Maj^+^(*G*)) *if and only if **and j *∈ *K*_*X*_(*G*).

Suppose we have a fill-in for *M*(*P*) that corresponds to some *G *= (*T*_1_,..., *T*_*k*_) ∈ ⟨*P*⟩_*r*_. Our ILP has two kinds of objective function variables. The first group of variables are denoted *w*_1_,..., *w*_*m*_, where *w*_*i *_corresponds to the *i*th column of *M*(*G*). Suppose this column corresponds to split *X *in tree *T*_*j*_; thus, *j *∈ *K*_*X*_(*G*). Our ILP has constraints such that *w*_*i *_= 1 if and only if . Thus, *w*_*i *_= 0 means that , which, together with Lemma 1 (ii), implies that .

The second group of variables are denoted *z*_*ij*_, 1 ≤ *i *≤ *m*, 1 ≤ *j *≤ *k*. Suppose column *i *of *M*(*P*) corresponds to split *X*. Our ILP has constraints such that *z*_*ij *_= 1 if and only if *w*_*i *_= 1 (i.e., ), *j *∈ , and *j *= min{ℓ : ℓ ∈ }. Thus, by Lemma 1 (i), .

The objective function can now be expressed as

#### Auxiliary variables and constraints

As mentioned earlier, all variables, including the auxiliary ones, are Boolean. We take advantage of this, expressing the constraints relating the variables as Boolean expressions in terms of the "and', "or," "exclusive or," and "if and only if" operators (denoted by the usual symbols, and, ∧, ∨, , and ⇔, respectively). We then convert these expressions into equivalent linear inequalities on zero-one variables using standard techniques [[30], pp. 231-244].

We first describe the variables and constraints that are used to ensure that the settings of the fill-in variables (the *F *variables) correspond to a restricted representative selection. That is, the assignments to the *F *variables must be such that, for each input tree *t*_*j*_, the resulting plenary splits associated with the tree are pairwise compatible, so that they yield a plenary tree *T*_*j *_∈ ⟨*t*_*j*_⟩_*r*_. For this purpose, we define variables *C*_*pq*_, 1 ≤ *p, q *≤ *m *and add constraints linking these variables and the *F *variables such that *C*_*pq *_= 1 if and only if columns *p *and *q *are compatible under the fill-in represented by the *F *variables. To guarantee that the assignment to the *F *variables corresponds to a restricted representative selection, we require that *C*_*pq *_= 1 for every two column indices *p, q *that correspond to splits in the same input tree. We note that the constraints relating the fill-in variables *F *and the *C*-variables closely resemble the ones used by Gusfield et al. [26]. One difference is that for our problem we need "if and only if" relationships, whereas Gusfield et al. require only one direction of the implication.

The constraints on the *C*-variables use the fact that splits *p *and *q *are incompatible if and only if 00, 01, 10, and 11 all appear in some rows of columns *p *and *q *(the "four gametes condition"). The presence or absence of these patterns for columns *p *and *q *is indicated by the settings of variables , *a, b *∈ {0, 1}, where  = 1 if and only if there is a taxon *r *such that *F*_*rp *_= *a *and *F*_*rq *_= *b*. The  are determined from the settings of variables , where *r *ranges over the taxa (i.e., the rows of *M*(*P*)). The Γ variables satisfy  ⇔ ((*F*_*rp *_= *a*) ∧ (*F*_*rq *_= *b*)). This condition is expressed by the following constraints.(3)

We have that , which is expressed by the inequalities below.(4)

Observe that . Equivalently we have the constraints below.(5)

We now consider the variables and constraints that enable us to express the objective function variables. There are three main sets of variables:

• For 1 ≤ *p *≤ *m*, *D*_*p *_equals 1 if and only if column *p *represents the same split as some column with smaller index.

• For 1 ≤ *i *≤ *m*, 1 ≤ *j *≤ *k*, , equals 1 if and only if split *i *is in tree *j*.

• For 1 ≤ *i *≤ *m*, 1 ≤ *j *≤ *k*,  equals 1 if and only if split *i *is compatible with tree *j*.

As we shall see, the values of the *w *and the *z *variables in the objective function are determined, respectively from the *S*^(1) ^and *S*^(2) ^variables, and from the *w*, *S*^(2)^, and *D *variables.

The *D *and *S*^(1) ^variables depend on variables *E*_*pq*_, 1 ≤ *p, q *≤ *m*, where *E*_*pq *_= 1 if and only if columns *p *and *q *of the filled-in matrix represent the same split. Here we have to make a distinction between rooted and unrooted trees. In the rooted case, there exists a root taxon *r *such that *M*_*ri*_(*P*) = 1 for every column *i*. The same is not true for unrooted trees.

The value of *E*_*pq *_depends on the patterns that appear in columns *p *and *q*, which can be deduced from the values of  for different choices of *a *and *b *as follows.

• For rooted trees, . This is expressed as follows.(6)

• For unrooted trees, we introduce two auxiliary variables  and  such that

Then,

These logical constraints are expressed by the following inequalities.(7)

We are now ready to give the constraints for the *D*, *S*^(1) ^and *S*^(2) ^variables. Observe that *D*_1 _= 0 and that, for 1 <*p *≤ *m*, . Equivalently we have(8)

In describing the constraints for the *S*^(1) ^and *S*^(2) ^variables, we adopt the convention that the splits of the *j*th tree correspond to columns *j*_1_,..., *j*_*d *_of *M*(*P*). Then, . This translates into the equality constraint(9)

On the other hand, . This is equivalent to the two constraints below.(10)

Finally, we describe how the objective function variables relate to the auxiliary variables. For each *i*, *w*_*i *_= 1 if and only if . This is expressed by the following two constraints.(11)

It follows from the definition of the *z *variables that, for every *i, j*, *z*_*ij *_⇔ *w*_*i *_∧ ¬ ∧ ¬*D*_*i*_. Equivalently we have the following.(12)

Table 1 summarizes the number of variables of each kind in our integer programming formulation. Here, as usual, *n *is the total number of taxa and *m *is the total number of splits in the input trees. As can be seen, there are a total of *O*(*nm*^2^) variables; this number is dominated by the Γ variables. The total number of constraints for the unrooted case, broken down by constraint type, is given by the following expression.

**Table 1:** Variables in the ILP formulation

*F*	Γ	*B*	*δ*^(*i*)^	*E*	*C*	*D*	***S***^(***i***)^	*w*	z
*mn*	2*m*(*m*-1)*n*	2*m*(*m*-1)	*m*(*m*-1)/2	2*m*(*m*-1)	2*m*(*m*-1)	*m*	*mk*	*m*	*mk*

The number of variables of each kind is expressed in terms of *n*, *m*, and *k*, the total number of taxa, the  total number of splits in the input trees, and the number of input trees, respectively.

The number of constraints for the rooted case is slightly smaller, but of the same order of magnitude. It should be noted that the expressions given in Table 1 assume that all the variables listed are indeed variables. In reality, the values of many of the *F *variables are fixed because they correspond to non-question-mark entries in *M*(*P*). This in turn fixes the values for several Γ variables, as well as those of other variables. As a consequence, the number of true variables in the ILP formulation is typically much smaller than the worst case estimates in Table 1. In general, the larger the number of question marks in matrix *M*(*P*), the closer the problem size will be to the worst case estimates.

#### Building Maj^+^(*P*)

The ILP model just outlined allows us to find a *G *∈ ⟨*P*⟩_*r *_corresponding to some optimal candidate supertree T*. To build Maj^+^(*P*) we need, in principle, the set of all such *G*. While there are ways to enumerate this set [31], we have found that an alternative approach works much better in practice. The key observation is that, since Maj^+^(*P*) is the strict consensus of all optimal candidate supertrees, each split in Maj^+^(*P*) must also be in T*. Thus, once we have T*, we simply need to verify which splits in T* are in Maj^+^(*P*) and which are not. To do this, for each split *A*|*B *in T*, we put additional constraints on the original ILP requiring that the optimal tree achieve an objective value equal or smaller than that of T* and *not *display split *A*|*B*. The resulting ILP has only *O*(*mn*) more variables and constraints than the original one. If the new ILP is feasible, then *A*|*B *∉ Spl(Maj^+^(*P*)); otherwise, *A*|*B *∈ Spl(Maj^+^(*P*)). We have found that detecting infeasibility is generally much faster than finding an optimal solution.

### A data reduction heuristic

The ILP formulation described in the previous section allows us to solve supertree problems of moderate size. Here we describe a data reduction heuristic that allows us to extend the range of our method significantly in practice, by exploiting the structure that is present in certain supertree problems. Our data reduction heuristic applies when the input profile *P *= (*t*_1_,..., *t*_*k*_) contains a subset of taxa *S *that can be treated as a single super-taxon. Roughly stated, we are looking for a set *S *such that every tree in *P *respects the split implied by *S*. We now define this concept more precisely.

Let Spl_0_(*T*) denote the set of *all *full splits displayed by *T*. That is, Spl_0_(*T*) includes the non-trivial and the trivial splits displayed by *T*; in particular, ℒ(*T*)|∅ ∈ Spl_0_(*T*). We say that *S *⊆ ℒ(*P*) with 1 < |*S*| < |ℒ(*P*)| -1 is a *reducible set *if, for each *j *∈ *K*, there is a split *A*|*B *∈ Spl_0_(*t*_*j*_) such that *A *∩ *S *= *A *and *B *∩ *S *= ∅. Ideally, a reducible set should correspond to a widely-acknowledged biological classification unit. For example, some of the trees in a collection of phylogenies may contain subtrees corresponding to different (possibly empty) subsets of the primates. While these subsets may not be identical, and the subtrees may disagree somewhat in their topologies, the input phylogenies are likely to separate primates from non-primates. In settings like this, it makes intuitive sense to restrict our attention to supertrees where reducible sets appear as clusters.

Given a reducible set *S *for *P*, we can define two smaller subproblems.

• The *reduced profile associated with a reducible set S *is the profile  where, for *k *each *j *∈ *K*,  is the tree obtained from *t*_*j *_by contracting the minimal subtree of *t*_*j *_containing *S *∩ ℒ(*t*_*j*_) to a single leaf node *β*_*S*_. If *S *∩ ℒ(*t*_*j*_) = ∅, then  = *t*_*j*_. We refer to *β*_*S *_as the *supertaxon associated with S*.

• The *satellite profile associated with S *is the profile  where  is obtained from *t*_*j *_by contracting the minimal subtree of *t*_*j *_containing (ℒ(*P*)\*S*) ∩ ℒ(*t*_*j*_) to a single leaf node *ρ*_*S*_. Note that some of the trees in the satellite profile associated with *S *may contain only *ρ*_*S*_. The *compressed satellite profile associated with S *is the satellite profile associated with *S *with all of the latter trees removed.

An *S-restricted *representative selection for *P *is a selection *R *= (*T*_1_,..., *T*_*k*_) ∈ ⟨*P*⟩ such that *S*|(ℒ(*P*)\*S*) ∈ Spl(*T*_*i*_) for all *i *∈ *K*. An *optimal S-restricted candidate representative selection *is an *S*-restricted representative selection *R *with minimum score, and Maj(*R*) an *optimal S-restricted candidate supertree*. The *S-restricted majority-rule (+) supertree *is the strict consensus of all the optimal *S*-restricted candidate supertrees.

**Figure 1:**
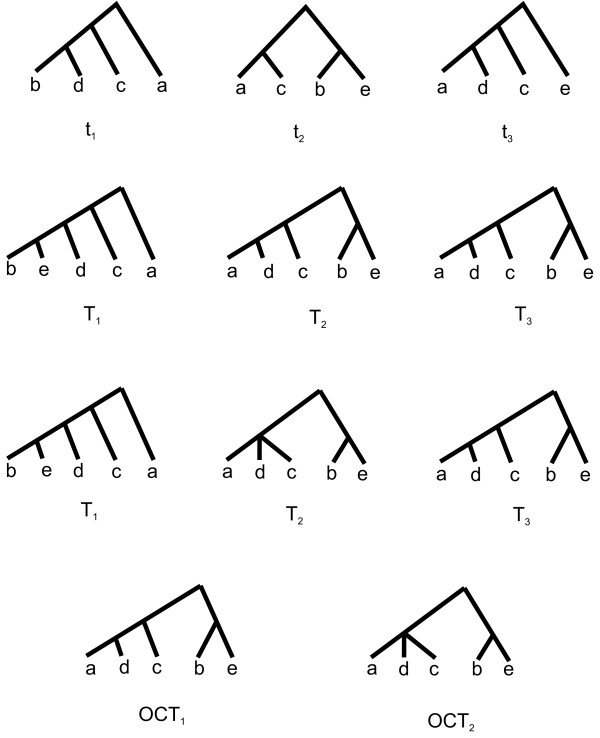
**Optimal candidate trees do not always display reducible sets**. Top row: An input profile *P *= (*t*_1_, *t*_2_, *t*_3_) where {*b*, *d*} and {*b*, *e*} are reducible sets. Second and third rows: Two optimal representative selections. Fourth row: Optimal candidate trees corresponding to the representative selections in rows two and three. Neither candidate tree contains the cluster {*b*, *d*}, although they both contain {*b*, *e*}.

It should be noted that, given an arbitrary reducible set *S*, it is not true in general that an optimal *S*-restricted candidate supertree will be an optimal candidate supertree, nor that an *S*-restricted majority-rule (+) supertree will also be a majority-rule (+) supertree. This is illustrated in Figure 1, which shows a profile *P *= (*t*_1_, *t*_2_, *t*_3_) where both {*b, e*} and {*b, d*} are reducible sets, but where neither optimal candidate tree contains the cluster {*b, d*}, although they both contain {*b, e*}.

On the other hand, a reducible set may represent useful biological knowledge that should be incorporated into a supertree analysis. There are also computational benefits. With the right choice of *S *(one where |*S*| is far from the extreme values of 2 and |ℒ(*P*)| -2), the reduced and satellite profiles can be considerably smaller than the original profile, and the corresponding integer programs will have fewer unknown variables. As the following theorem indicates, an optimal *S*-restricted candidate supertree can be found by solving the associated subproblems separately and combining their answers.

**Theorem 5**. *Let P be a profile and S be a reducible set in P. Let T*^*Red *^*and T*^*Sat *^*be optimal candidate trees for the reduced profile associated with S and the compressed satellite profile associated with S. Let T be the tree obtained by identifying the node β*_*S *_*in T*^*Red *^*and node ρ*_*S *_*in T*^*Sat *^*and then suppressing the resulting degree-two vertex. Then, T is an optimal S-restricted candidate supertree for P. Further, if R, is the optimal S-restricted representative selection corresponding to T and R*^*Red *^*and R*^*Sat *^*are the optimal representative selections corresponding to T*^*Red *^*and T*^*Sat*^, *respectively, then s*(*R*) = *s*(*R*^*Red*^) + *s*(*R*^*Sat*^).

The straightforward proof of this result is omitted. A direct consequence is that the *S*-restricted majority-rule (+) supertree can be obtained by piecing together the majority-rule (+) supertrees for the reduced and satellite profiles. Observe that if multiple pairwise disjoint reducible sets are known, then each of the corresponding compressed satellite profiles can be solved independently, and the original profile can be reduced by replacing each reducible set to a distinct supertaxon. In fact, the idea can be used recursively, so that a satellite profile can itself be decomposed to a reduced profile and (sub) satellites. As we shall see later, this can result in dramatic problem size reductions.

## Results and discussion

Here we report on computational tests with the exact ILP method and the data reduction heuristic. All our experiments were conducted on real data sets, rather than simulated data. We did this because we were interested in seeing if the groupings of taxa generated by majority-rule (+) supertrees would coincide with those commonly accepted by biologists. Another goal of our experiments was to compare the performance of the ILP formulation without data reduction, which we refer to as the *basic *method, against that of ILP plus data reduction. All trees considered in our tests were rooted.

To conduct our tests of the basic method, we wrote a program to generate the ILPs from the input profiles. For our tests of the data reduction heuristic, we used different methods to find reducible sets in a profile; these are outlined later. Given the reducible sets, the corresponding reduced and satellite profiles, as well as the associated ILPs, were generated automatically. All ILPs were then solved using CPLEX (CPLEX is a trademark of ILOG, Inc.) on an Intel Core 2 64 bit quad-core processor (2.83 GHz) with 8 GB of main memory and a 12 MB L2 cache per processor.

### Experiments with the basic ILP formulation

We tested the basic ILP formulation on five published data sets. The *Drosophila A *data set is the example studied in [14], which was extracted from a larger Drosophila data set considered by Cotton and Page [32]. *Primates *is the smaller of the data sets from [5]. *Drosophila B *is a larger subset of the data studied in [32] than that considered in [14]. *Chordata A and B *are two extracts from a data set used in a widely-cited study by Delsuc et al. [33]. Chordata A consists of the first 6 trees with at least 35 taxa (out of 38). Chordata B consists of the first 12 trees with at least 37 taxa (out of 38).

The results are summarized in Table 2. Here *n*, *m*, and *k *are the number of taxa, total number of splits, and number of trees, respectively. *U *denotes the number of question marks in *M*(*P*), the matrix representation of the input; *N *is the size of the CPLEX-generated reduced ILP. Table 2 shows the time to solve the ILP and produce an optimal candidate supertree T* and the time to verify all the splits of T* to produce Maj^+^(*P*).

**Table 2:** Summary of experimental results with the basic ILP method

Data set	*n*	*m*	*k*	*U*	%*U*	*N*	Sol. (sec)	Verif. (sec)
Drosophila A	9	17	5	60	39.2	9.8 e5	0.83	1.6
Primates	33	48	3	590	37.3	7.8 e7	15.83	2.86
Drosophila B	40	55	4	1133	51.5	1.25 e9	362	19
Chordata A	38	290	6	330	3	1.40 e8	120	258
Chordata B	38	411	12	306	2	1.05 e8	986	1784

Size and solution times for the ILP formulations of the various data sets. Here *U *denotes the number of question marks in *M*(*P*), the matrix representation of the input; *N *is the size of the CPLEX-generated reduced ILP; *n*, *m*, and *k *are as in Table 1. Shown are the time to solve the ILP and produce an optimal candidate supertree T* and the time to verify all the splits of T* to produce Maj^+^(*P*).

### Experiments with the data reduction heuristic

As a preliminary test, we compared the results obtained via the reduction heuristic with the exact solutions, obtained using the basic ILP method, for two of the data sets listed in Table 2. For simplicity, only clusters from the input trees were used as reducible sets. (Note that unions of input clusters could have also been used as reducible sets.) We wrote a program that chooses clusters greedily. At every step, it selects the largest non-trivial cluster present in some input tree that does not overlap with any of the previously chosen clusters.

For the Primates data set, the optimal objective value (i.e., the score of an optimal candidate supertree) for the original profile is 9. We found six pairwise disjoint reducible sets, and built the corresponding reduced and satellite profiles. The optimal objective values of the reduced profile, first, second and third satellite profiles are 0, 4, 3, and 2, respectively. The other satellite profiles have an optimal objective value of 0. Thus, the total score of the reduced and satellite profiles matches the optimal score for the original profile, and the supertree obtained using the heuristic is also optimal. The reduction method also gives a correct optimal candidate supertree for Drosophila B. Here the original profile has an objective value of 8. We found nine pairwise disjoint reducible sets, and built the corresponding reduced and satellite profiles. The reduced profile has an optimal objective value of 8 and all satellite profiles have an optimal objective value of 0.

It should be pointed out that the reducible sets used for Primates and Drosophila B do not necessarily correspond to clusters in the majority-rule (+) supertree, although they are displayed by some optimal candidate trees. Thus, one will not obtain a majority-rule (+) supertree by simply composing the solutions to the reduced problems and the satellites. This indicates the importance of choosing relatively few large and well-supported reducible sets. Biological knowledge can serve as a good guide. For example using the clade *Haplorrhini *as a reducible set for Primates data set, solving the corresponding reduced and satellite profiles and combining the respective majority-rule (+) supertrees one gets exactly the same supertree as through the basic (and exact) method. Similarly, using the subgenus *Sophophora *as a reducible set for Drosophila B, we, obtained precisely the majority-rule (+) supertree for the data set.

Next, we considered some data sets that are well beyond the reach of our basic ILP method. The *Drosphila C *data set is the full 6-tree Drosophila data set of Cotton and Page [32] from which the Drosophila A and B data sets were extracted. The *Seabirds *data set consists of the 7 trees in the seabirds study by Kennedy and Page [17]; which encompasses 122 taxa (note that one of these taxa is an outgroup, so we do not count it in our study). We also examined the full Chordata set of Delsuc et al. [33], which has 38 taxa and 146 trees.

#### Chordata

We looked for reducible sets in the full Chordata data set by considering increasingly larger subprofiles, starting with one input tree and then including one more input tree at every step. For each subprofile, we conducted an exhaustive search for reducible sets. The number of reducible sets increased at first, then fluctuated, and finally declined. After the 20th tree, there were no reducible sets. Thus, the data reduction heuristic proved to be ineffective for this data set.

#### Drosophila C

We identified seven reducible sets for Drosophila C. Six of these were found by the greedy approach; the seventh corresponded to the subgenus *Sophophora *(the latter was selected manually, after some of the subproblems identified by our program proved impossible to solve). Four of the associated satellites were trivially solvable, since each contained only two taxa. We then solved ILPs for the reduced and the nontrivial satellites. The running time statistics are summarized in Table 3, which shows the same kind of data shown in Table 2, except that this time it reports these statistics for the original, reduced and satellite problems. Notably, even though the original ILP was too large to be solved, the reduced profile was solved in less than 10 minutes and the satellite profiles were solved almost instantly.

**Table 3:** Results of Drosophila C analysis using data reduction

Data set	*n*	*m*	*U*	%*U*	*N*	Sol. (sec)	Verif. (sec)
Original	46	70	1998	62.1	9.4e9	N/A	N/A
Reduced	17	33	264	47.06	1.7e7	543.16	50.4
Satellite 1	17	17	146	50.52	2.3e6	0.23	0.28
Satellite 2	6	4	0	0	0	0.00	0.00
Satellite 3	5	3	0	0	0	0.00	0.02

Size and solution times for all six trees in trees in Cotton and Page's Drosophila data set [32] without and with data reduction. The notation is the same as in Tables 1 and 2. Shown are the time to solve the ILP and produce an optimal candidate supertree, and the values of the objective function for the reduced and satellite profiles. The latter two values are not available (N/A) for the original profile, since the model was too big to be solved. Not listed in the table are four other trivially solvable two-taxon satellite profiles.

#### Seabirds

To handle the Seabirds data set, we identified three reducible sets, which yielded a reduced profile and three satellite profiles, numbered 1, 2, and 3. Satellite profile 3 was too big to be solved by the basic ILP method, so it was further reduced by identifying three reducible sets within it, which resulted in three (sub-) satellite profiles, numbered 3.1, 3.2, and 3.3. The various reducible sets correspond to biologically meaningful classification units, as we explain next. In what follows, we refer to the 7 input trees of Kennedy and Page's seabirds data set by the same letters A-G that those authors used in [17].

Satellite 1 comprises the family *Spheniscidae *(Penguins, 10 taxa), which agrees with widely-accepted classifications for seabirds [34]. Members of this family appear in input trees E, F, and G of [17], and clearly form clusters of their own. Satellites 2 and 3 correspond to *Diomedeinae *(Albatrosses, 22 taxa), and *Procellariinae *(gadfly petrels, shearwaters, fulmars and diving petrels, 73 taxa). This agrees with the Sibley-Ahlquist classification [35] (represented by tree G). The resulting reduced profile has 19 taxa (16 original taxa and three supertaxa).

Satellite 3 (*Procellariinae*) has three subsatellites. Satellite profile 3.1 comprises the genus *Pterodroma *(30 taxa). Satellite 3.2 is for genus *Pelecanoides *(four taxa). Satellite 3.2 is a combination of *Puffinus *and *Calonectris *(10 taxa), which is supported by [36] (tree E). With these three sub-satellites, the reduced *Procellariinae *profile has 23 taxa (20 original taxa and three supertaxa).

**Figure 2:**
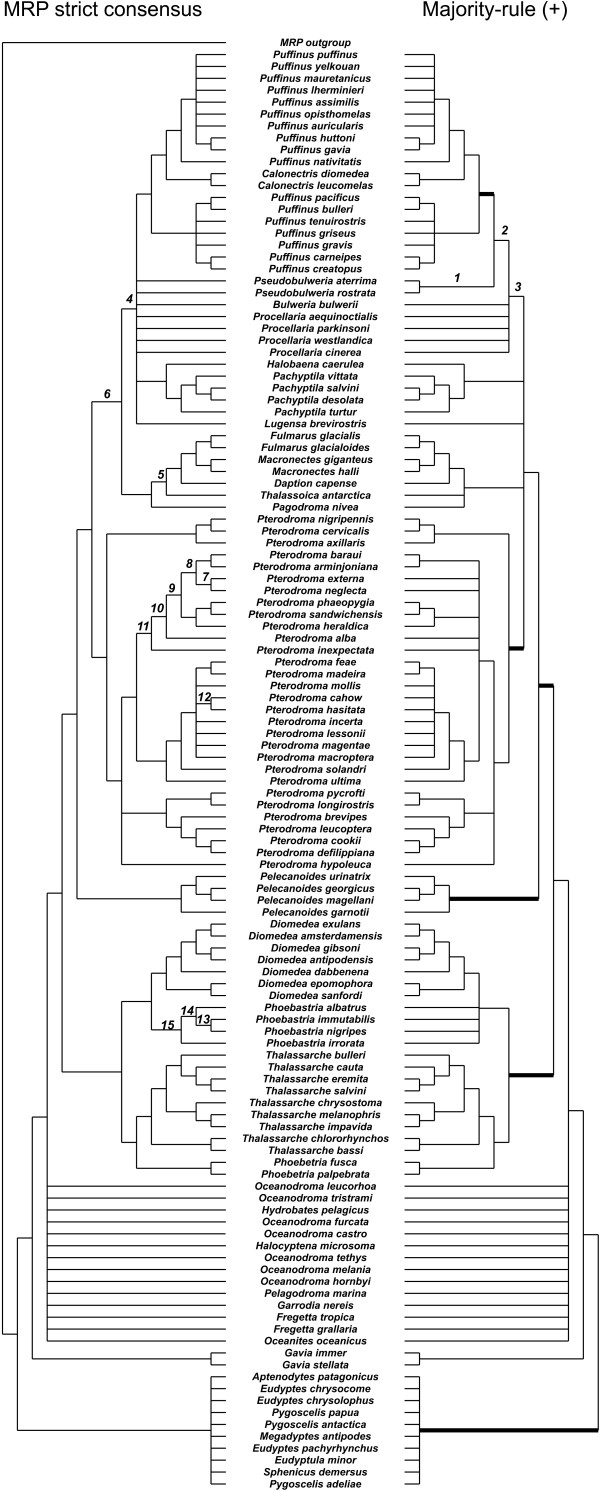
**Comparing the MRP strict consensus with the majority-rule (+) supertree**. Left: The strict consensus of the most parsimonious trees obtained by Kennedy and Page for their seabirds data set [17]. Right: The majority-rule (+) supertree obtained by using the data reduction heuristic; the reducible sets used to construct it are indicated by heavy lines. Clusters that appear in one tree but not the other are numbered; the differences are explained in the text.

Table 4 summarizes the results on the Seabirds data set. The majority-rule (+) supertree is shown in Figure 2, along with the MRP strict consensus tree of [32]. While the original problem was too big for CPLEX to solve on our machine, the reduced model was solved in 6.5 seconds. Most subproblems were solved and verified in a negligible amount of time. A notable exception was the reduced version of satellite 3, which required almost a minute to solve and nearly one hour and 45 minutes to verify.

**Table 4:** Results of Seabirds analysis using data reduction

Data set	*n*	*m*	*U*	%*U*	*N*	Sol. (sec)	Verif. (sec)
Original	121	188	12620	55.4	2.63e12	N/A	N/A
Reduced	19	24	188	41.2	7.1e6	6.51	156.2
Sat. 1	10	8	42	52.5	1.1e5	0.05	0.07
Sat. 2	22	29	129	20.2	1.2e6	0.09	1.06
Satellite 3 (reduced)	23	39	370	41.3	5.1e7	52.3	6110
Subsatellite 3.1	30	42	196	15.6	6.8e5	0.06	0.04
Subsatellite 3.2	4	2	0	0	0	0.00	0.00
Subsatellite 3.3	19	20	113	29.7	9.5e5	0.17	0.06

Size and solution times for all seven trees in Kennedy and Page's seabirds data set [17] without and with data reduction. The notation is the same as in Table 3. The solution and verification times for the original profile are not available (N/A), since the model was too big to be solved.

### Discussion

Our results using the basic ILP formulation compare well with the published ones. For Drosophila A we obtained exactly the same tree reported in [14]. For Primates, the output is exactly the same as [5], which was produced by PhySIC method. The coincidence with PhySIC is noteworthy, since this supertree is less controversial than the MRP, Mincut, and PhySIC_PC_ supertrees reported in [5]. The reason for the coincidence may lie in the fact that, while heuristic, PhySIC requires that all topological information contained in the supertree be present in an input tree or collectively implied by the input trees, which bears some similarity with properties (CW1)-(CW4) of majority (+) supertrees.

For Drosphila B, Cotton and Page [32] show four supertrees: strict consensus of gene tree parsimony (GTP), Adams consensus of GTP, strict consensus of MRP, Adams consensus of MRP. Among the 10 clusters found by our ILP, two are in all four of these supertrees, three are found in the Adams consensus of GTP and Adams consensus of MRP, one is in the strict and Adams consensus of GTP, and one is found in the strict and Adams consensus of MRP. Thus, with only four input trees we were able to generate a tree that is quite similar to the published results. For Chordata A, the 12 splits found matched published results [33] exactly. For Chordata B, the 14 splits found matched [33].

We have not mapped out the precise boundary within which it is feasible to use the basic ILP method. However, it appears that it may not extend much beyond the dimensions of the problems listed in Table 2. For example, Drosophila B contains four out of 6 of the trees studied in [32]. Adding a fifth tree to the data set yields a problem that could not be solved by the basic ILP method. A major factor here is that the size of our ILP grows as the square of the total number of splits in all trees, and the solution time is exponential in the worst case. Incorporating a new tree to Drosophila B could easily add enough splits to the problem to put it well beyond the reach of our technique. We should add that model size does not appear to be the sole factor that makes instances hard — sparsity also seems to play a role.

#### Drosophila C

The majority-rule (+) supertree for Drosphila C constructed by our method (available upon request) has 15 nontrivial clusters, while the MRP strict consensus tree of Cotton and Page [32] has 11. Of these only three appear in both trees. This rather surprising result motivated us to try to assess how well the input trees are represented by the supertree. To this end, we relied on the notions of support and conflict, along the lines proposed by Wilkinson et al. [37].

Let *t *be an input tree for a profile *P*, *T *be a supertree for *P*, and *S *be a non-trivial cluster in *T *(i.e., *S *does not contain the root of *T *and *S|*(ℒ(*P*)\*S*) ∈ Spl(*T*)). Let *S' *= *S *∩ ℒ(*t*). We say that tree *t supports S *if *S' *is a non-trivial cluster in *t*. Tree *t *is *in conflict with S *if *S' *is incompatible with *t*; i.e., there is no tree *t' *with ℒ(*t'*) = ℒ(*t*) such that Spl(*t*) ∪ {*S'*|(ℒ(*t*)\*S'*)} ⊆ Spl_0_(*t'*). If *t *neither supports nor is in conflict with *S*, we say that *t *is *irrelevant *to *S*.

Theorem 1 hints that each cluster *S *in the majority-rule (+) supertree should have more input trees supporting it than contradicting it, even when most trees are irrelevant to *S*. This indeed holds for the Drosophila C majority-rule (+) supertree: Every one of its non-trivial clusters is supported by at least one input tree and does not conflict with any input tree. In contrast, of the five clusters in the MRP strict consensus supertree for which support outweighs conflict, only three have no conflict with any input tree. Of the remaining clusters, three have the same amount of conflict as support, and for three others the amount of support is outweighed by the amount of conflict. In fact, among the latter, there is a cluster that is in conflict with five out of six of the input trees; the remaining tree is irrelevant to that cluster. We refrain from claiming the superiority of one supertree over the other, since the biological relevance of both trees needs to be studied in more detail.

#### Seabirds

Figure 2 compares the majority-rule (+) supertree for the seabirds data set, constructed using the data reduction heuristic, with the MRP strict consensus supertree that Kennedy and Page presented for the same data set [17]. The latter is the strict consensus of 10,000 equally parsimonious trees obtained using MRP. There are 66 nontrivial clusters in the majority-rule (+) supertree, compared with 75 nontrivial clusters in the MRP strict consensus tree (ignoring the outgroup). Among these clusters, 63 are present in both trees (95% of 66 and 84% of 75). The reducible sets used to construct the majority-rule (+) supertree are indicated by heavy lines. Note that these sets are also clusters in the MRP supertree.

Three clusters, numbered 1-3 in Figure 2, are in the majority-rule (+) supertree but not in the MRP tree; 12 clusters, numbered 4-15 in Figure 2, appear in the MRP tree but not in the majority-rule (+) tree. For each of the seven input trees (labeled A-G in [17]) and each of these 15 clusters, Table 5 indicates whether the tree supports, is in conflict with, or is irrelevant to the cluster. As Theorem 1 would lead us to expect, each of clusters 1-3 (from the majority-rule (+) tree) has more input trees supporting it than in conflict with it. Of the 12 clusters (4-15) that are present only in the MRP strict consensus tree, seven have as many trees in support as in conflict. The others have more support than conflict.

**Table 5:** Support and conflict for the Seabirds data set

	Input Tree						
	
Cluster	A	B	C	D	E	F	G
1	i	s	i	i	i	i	i
2	s	s	s	i	s	s	s
3	s	s	s	i	c	s	s
4	s	s	s	i	s	c	c
5	i	s	s	i	s	i	c
6	i	s	s	i	s	c	c
7	i	i	i	s	c	i	i
8	i	i	i	s	c	i	i
9	i	i	i	s	c	i	i
10	i	i	i	s	c	i	i
11	i	i	i	c	s	i	i
12	i	i	i	s	c	i	i
13	i	i	i	i	s	i	c
14	i	i	s	i	s	i	c
15	i	i	s	i	s	i	c

For each of the 15 clusters that is present in only the majority-rule (+) supertree or only the MRP strict consensus supertree of the Seabirds data set, we indicate whether each of input trees A through G supports (s), is in conflict with (c), or is irrelevant to (i) the cluster. The numbering of the clusters follows Figure 2.

In general, it appears that MRP may have a bias toward preserving clusters that are present in trees that contain many members of the families represented in those clusters. This is noticeable for *Pterodroma*, where the disagreement between trees D and E is resolved in favor of the former five times to one, in clusters 7, 8, 9, 10, and 12 versus cluster 11. This may be related to the "size bias" that previous researchers have observed in MRP [38]: Here, even though E is the larger tree (90 taxa versus 30), D has more taxa in the *Pterodroma *genus (30 versus 16). Majority-rule (+) trees seem not to have such a bias, because the expansion process used to construct representative selections tends to put all input trees, regardless of their size, on equal footing. These are, of course, only preliminary observations; this issue clearly deserves further analysis.

## Conclusions

Our results indicate that the majority-rule (+) method produces biologically reasonable phylogenies (i.e., phylogenies with no unsupported groups), and that the method is practical for medium-scale problems. Unfortunately, while polynomial, the size of our ILP is quadratic in the total number of splits in the input trees. This, together with the fact that solving the ILP takes exponential time in the worst case limits the range of applicability of the basic ILP formulation. It also explains in part why the addition of a single tree to a data set can convert a tractable problem into an intractable one. More extensive tests are needed to assess the limitations of the basic ILP approach accurately. In any event, our computational experience shows that the technique does handle some real, biologically significant, problems nicely. Moreover, our results suggest that the ILP approach, in combination with our data reduction heuristic is a promising way to tackle larger problems.

## Competing interests

The authors declare that they have no competing interests.

## Authors' contributions

JD developed the methods, programmed them, conducted the computational experiments, and wrote the first draft of the manuscript. DFB supervised the work of JD, and contributed to the method development, the experimental design, and to the writing of the manuscript. FRMcM contributed to the theoretical foundations of the method, especially to the formulation and proof of Theorem 1; he also contributed to the writing of the manuscript.
